# Integrative Analyses of Long Non-coding RNA and mRNA Involved in Piglet Ileum Immune Response to *Clostridium perfringens* Type C Infection

**DOI:** 10.3389/fcimb.2019.00130

**Published:** 2019-04-30

**Authors:** Xiaoyu Huang, Wenyang Sun, Zunqiang Yan, Hairen Shi, Qiaoli Yang, Pengfei Wang, Shenggui Li, Lixia Liu, Shengguo Zhao, Shuangbao Gun

**Affiliations:** ^1^College of Animal Science and Technology, Gansu Agricultural University, Lanzhou, China; ^2^College of Life Science and Engineering, Northwest Minzu University, Lanzhou, China; ^3^Gansu Research Center for Swine Production Engineering and Technology, Lanzhou, China

**Keywords:** piglet, lncRNA, mRNA, *Clostridium perfringens* type C, ileum, immune response

## Abstract

Long non-coding RNAs (lncRNAs) have been shown to play important roles in regulating host immune and inflammatory responses to bacterial infection. Infection with *Clostridium perfringens* (*C. perfringens*), a food-borne zoonotic pathogen, can lead to a series of inflammatory diseases in human and piglet, greatly challenging the healthy development of global pig industry. However, the roles of lncRNAs involved in piglet immune response against *C. perfringens* type C infection remain unknown. In this study, the regulatory functions of ileum lncRNAs and mRNAs were investigated in piglet immune response to *C. perfringens* type C infection among resistance (IR), susceptibility (IS) and sham-inoculation (control, IC) groups. A total of 480 lncRNAs and 3,669 mRNAs were significantly differentially expressed, the differentially expressed lncRNAs and mRNAs in the IR and IS groups were enriched in various pathways of ABC transporters, olfactory transduction, PPAR signaling pathway, chemokine signaling pathway and Toll-like receptor signaling pathway, involving in regulating piglet immune responses and resistance during infection. There were 212 lncRNAs and 505 target mRNAs found to have important association with *C. perfringens* infectious diseases, furthermore, 25 dysregulated lncRNAs corresponding to 13 immune-related target mRNAs were identified to play potential roles in defense against bacterial infection. In conclusion, the results improve our understanding on the characteristics of lncRNAs and mRNAs on regulating host immune response against *C. perfringens* type C infection, which will provide a reference for future research into exploring *C. perfringens*-related diseases in human.

## Introduction

*Clostridium perfringens* (*C. perfringens*) is a Gram-positive anaerobic rod and ranks as the second most common bacteria that causes fulminant, fatal infectious and immune diseases (Scharff, 2012; Grass et al., 2013). These diseases are characterized by fever, pain, gas production, local edema, and severe tissue destruction, they usually further develop into systemic toxemia, necrotic enteritis, shock, sepsis, or even death in many animal species, including human and piglet (Songer et al., 2010), which are estimated one million illnesses reported every year (Low et al., 2018). Considering the four major lethal toxins produced by *C. perfringens* (alpha, beta, epsilon, and iota), it has been subdivided into five types (A to E) (Hassan et al., 2015). *C. perfringens* infection in pigs is mainly caused by the type C strain, the infection in younger piglets (<1 week of age) usually results in enteric inflammatory diseases, which have a high mortality rate; in older pigs, it results in asymptomatic intestinal inflammation with persistent shedding of the organisms in feces. The economic loss, in general, has severely crippled development of the global pig industries (Chan et al., 2012). Therefore, reducing the incidence and severity of *C. perfringens* infectious diseases (CPID) is an urgent problem that needs to be resolved.

Pigs are the important transmitter and reservoirs of *C. perfringens* type C, the colonization and shedding of this bacterium occurs within infected or asymptomatic pigs, posing a huge risk to herd and public health (Kich et al., 2014). Controlling the transmission of *C. perfringens* type C from pigs to humans, through the environment and pork products, and simultaneously decreasing the prevalence of *C. perfringens* type C in pig litters and herds may effectively reduce CPID in humans, especially in large pork-producing and consuming countries. According to the bacteria's pathogenicity, the host's immune activities and the cross-action between them, hosts generally manifest different severities of responses to bacterial infection (Zanella et al., 2011), which resulted in the dynamic profiles of host resistance/susceptibility to specific pathogen. Enhancing the disease resistance of host may serve as a useful alternative approach to control the spread of *C. perfringens* type C.

Long non-coding RNA (lncRNA) is a type of nucleotide transcripts, longer than 200 nucleotides in length, without coding potential. The lncRNAs can participate in regulation of mRNA expression, through their abilities to activate or restrain protein coding genes (Engreitz et al., 2016). Researches have clearly demonstrated that the various lncRNAs have participated in the development of immune system and in regulation of host inflammatory response against pathogenic bacteria (Ding et al., 2016). For example, *Salmonella* infection has been shown to cause changes in expression of certain sensitive lncRNAs in the early stage of infection in HeLa cells (Westermann et al., 2016). The long intergenic non-coding RNA (lincRNA)-cyclooxygenase 2 (*Cox2*) has been shown to regulate transcription of inflammation-related genes in the innate immune response through the NF-κB signaling pathway, and *Listeria monocytogenes* infection has been shown to cause the up-regulation of lincRNA-*Cox2* in macrophages (Carpenter et al., 2013). The lncRNA p50-assocated Cox-2 extragenic RNA (known as *PACER*) has been shown to be up-regulated in LPS-infected human macrophages, subsequently regulating expression of prostaglandin endoperoxide synthase 2 (*PTGS2*) via the NF-κB signaling pathway (Krawczyk and Emerson, 2014). LncRNA HOX transcript antisense RNA (*HOTAIR*), a positive regulator of inflammation, has been shown to induce production of TNF-α by NF-κB activation, to regulate infection response in lipopolysaccharide (LPS)-induced sepsis (Wu et al., 2016). The up-regulation of lncRNA TCONS00183659 in resistant piglets has been shown to regulate the expressions of inflammatory factors *MX1, MX2*, and *IFIT2*, through combining with histone H4, to improve the resistance of weaning piglet defense against *Escherichia coli* (*E. coli*) F18 infection (Wu, 2018). In addition, in the *E. coli* F18-infected mice diarrhea model, the up-regulation of lncRNA ENSMUST00000122226 has been shown to improve the expression of adhesion molecule CD28 on T cell surface to facilitate the secretion of various inflammatory cytokines, including IFN and IL, finally leading intestinal inflammatory response and diarrhea disease (Zhao, 2016). While these collected studies have highlighted the array of dynamic changes and roles of lncRNAs in regulating host immune and inflammatory responses during bacterial and viral infections, the molecular interaction and regulatory roles of lncRNAs involved in host immune responses to *C. perfringens* type C infection have not yet been defined.

The intestine serves as the important physical barrier to prevent systemic invasion of pathogen. Invasion of pathogen in the intestine first triggers a series of antibacterial responses of immune cells residing in the tissue, the interactions between the two manifest the resistance capacities of each (the invading pathogen and the responding host). To evaluate functions of lncRNAs and mRNAs in regulating piglet immune responses to *C. perfringens* type C, we performed a comprehensive analysis of the expression profiles of lncRNAs and mRNAs in ileum of *C. perfringens* type C-infected piglets using RNA sequencing, the results could provide support for the merit of exploring molecular mechanisms of lncRNAs and mRNAs underlying a mammalian immune response to the *C. perfringens* infection.

## Materials and Methods

### Preparation of *C. perfringens* Type C Strain

The *C. perfringens* type C strain (CVCC 2032) was obtained from the China Veterinary Culture Collection Center (Beijing, China). The bacteria were cultured in bouillon culture-medium (HopeBio, Qingdao, China) under anaerobic conditions with 5% (v/v) H_2_: 5% (v/v) CO_2_ and 90% (v/v) N_2_ mixtures, at 37°C for 16 h with shaking before infection. The numbers of colony-forming units (CFUs) of *C. perfringens* type C were determined by plate colony counting method, and an expected concentration of 1 × 10^9^ CFU/mL *C. perfringens* type C medium was used to inoculate piglets.

### Sample Collection

Experimental piglets were from a *C. perfringens* seronegative Landrace × Yorkshire healthy nucleus herd in Dingxi, Gansu, China. Blood samples were collected from every piglet via anterior vena cava and into sterile coagulation-promoting tubes. Serum samples were obtained after centrifugation at 2,000 × g for 10 min at 4°C, and then confirmed antibody-negative for detection of porcine *E. coli, Salmonella*, and *C. perfringens* by the commercial enzyme-linked immunosorbent assay kits (Jiancheng Bioengineering Institute, Nanjing, China). Finally, thirty 7-day-old piglets (15 males and 15 females) were selected and randomly assigned into two groups (inoculated group: *n* = 25; control group (IC): *n* = 5). Piglets in the inoculated group were orally inoculated with 1 mL 1 × 10^9^ CFU/mL *C. perfringens* type C medium for 5 consecutive days, while piglets in the IC group received orally sham-inoculation with sterile culture media. All piglets were separately housed and maintained in a climate-controlled and fully isolated environment, with water and diets provided *ad libitum*. After infection, piglets were monitored twice per day for general health, behavior, appetite, body condition, hair coat and dehydration. Fecal consistency was evaluated 4–5 times daily, and scored based on the visual symptom traits (Yang et al., 2013; Huang et al., 2016): 0 = normal, solid feces; 1 = slight diarrhea, soft and loose feces; 2 = moderate diarrhea, semi-liquid feces; 3 = severe diarrhea, watery feces.

Grouping were made as follows: firstly, recording fecal consistency of every defecation of each piglet, then calculating and ranking the total fecal scores of every piglet, and lastly, defining the top five piglets with highest and lowest fecal scores as the susceptibility (IS) and resistance (IR) groups, respectively. A total of 15 piglets from IR, IS and IC groups were humanely euthanized at 6 days post-challenge (dpc). Ileum tissues were collected and flushed clean with sterile PBS buffer (pH 7.4), and then quickly frozen in liquid nitrogen and stored at −80°C until RNA extraction.

### Effect of *C. perfringens* Type C Infection on Number of Bacteria in Feces

The fecal samples were collected by sterile rectal swab every day and serially diluted in sterile PBS. From each dilution, 100 μL sample was plated in triplicate on 100 mL tryptose sulfite cycloserine (TSC) agar base supplemented with 10 mL D-cycloserine and 10 mL egg yolk emulsion (HopeBio, Qingdao, China). Plates were anaerobically incubated at 37°C for 24h. The fecal *C. perfringens* type C CFUs in feces of piglets from the IR, IS, and IC groups were compared using the Student's *t*-test in SPSS 22.0 software (IBM Corp., Armonk, NY, USA). The *P* < 0.05 was considered statistically significant.

### Total RNA Isolation

Total RNA was isolated from each individual sample using Trizol™ reagent (Invitrogen, Carlsbad, CA, USA). RNA concentration was measured using Qubit® RNA Assay Kit in Qubit® 2.0 Flurometer (Life Technologies, Frederick, MD, USA). Purity and integrity of total RNA were assessed using the NanoPhotometer® spectrophotometer (IMPLEN, Westlake Village, CA, USA) and RNA Nano6000 Assay Kit of the Agilent Bionalyzer 2100 system (Agilent Technologies, Palo Alto, CA, USA). Sample with the RNA integrity number (RIN) > 7.0 were used for library preparation.

### Library Preparation and RNA-Seq Data Acquisition

Approximately 3 μg total RNA per sample was used for preparing RNA sequencing libraries. Ribosomal RNA (rRNA) was removed from total RNA by epicenter Ribo-zero™ rRNA Removal Kit (epicenter, USA), and rRNA free residue was cleaned up by ethanol precipitation. Subsequently, rRNA-depleted RNA (Ribo-Zero RNA) was used to generate strand-specific RNA sequencing libraries by NEBNext® Ultra™ Directional RNA Library Prep Kit for Illumina® (NEB, Ipswich, MA, UK), which can capture all transcripts with and without poly A. To select cDNA fragments of preferentially 150–200 bp in length, the library fragments were purified with AMPure XP system (Beckman Coulter, Beverly, USA). Then 3 μL USER Enzyme (NEB, USA) was used with size-selected, adaptor-ligated cDNA at 37°C for 15 min followed by 5 min at 95°C before PCR, then PCR reaction was performed using Phusion High-Fidelity DNA polymerase, Universal PCR primers and Index (X) Primer. At last, products were purified (AMPure XP system) and library quality were assessed on the Agilent Bioanalyzer 2100 system.

The clustering of the index-coded samples was performed on a cBot Cluster Generation System using the TruSeq PE Cluster Kit v3-cBot-HS (Illumina®). After cluster generation, RNA libraries were sequenced on an Illumina Hiseq 4000 platform (Illumina, San Diego, CA, USA) to generate 150bp paired-end (PE150) reads at the Novogene Bioinformatics Institute (Beijing, China).

### Transcriptome Assembly

Firstly, clean reads were obtained by discarding reads that contained adapter, ploy-N and low quality (>50% of bases with Phred scores <5) reads from raw data (fastq format) processed through in-house perl scripts. Simultaneously, the Phred score (Q20, Q30), and GC content of the clean data were calculated. Secondly, the paired-end clean reads were mapped to the pig reference genome sequence (*Sus scrofa* 10.2) by Tophat v2.0.9 (Kim et al., 2013). Lastly, the mapped reads of every sample were assembled by Scripture (Guttman et al., 2010) and Cufflinks (Trapnell et al., 2010) in a reference-based approach.

### Analyses of Coding Potential and Conservation

We used four tools to distinguish mRNA from lncRNA, namely Coding-Non-Coding-Index (CNCI) (Sun et al., 2013), Coding Potential Calculator (CPC) (Kong et al., 2007), Pfam-scan v1.3 (*E*-value < 0.001) (Punta et al., 2012), and phylogenetic codon substitution frequency (phyloCSF) (Lin et al., 2011), respectively. Transcripts with coding potential predicted by any one of these four tools were filtered out, and those without coding potential were filtered as the candidate set of lncRNA.

The Phast software (version 1.3) was generally used for phylogenetic analysis (Siepel et al., 2005) and PhastCons is a conservation scoring and identifying program of conserving elements. We used phyloFit to compute phylogenetic models for conserved and non-conserved regions and then set the model and HMM transition parameters for phyloP to calculate the conservation scores of lncRNA and coding genes.

### Prediction of lncRNA Target Genes

*Cis* and *trans* analyses were used to predict the target genes of differentially expressed lncRNAs. The *cis* role of lncRNAs indicated their actions on neighboring target genes, the coding genes close to 10 k upstream and downstream regions of lncRNA were considered as the *cis* role target genes (Guil and Esteller, 2012). The target genes of lncRNA in *trans* role were identified by expression levels, according to Pearson's correlation coefficient (|*r*| > 0.95).

### Identification of Differentially Expressed lncRNA and mRNA

The FPKMs (fragments per kilo-base of exon per million fragments mapped) of lncRNA and mRNA were calculated by Cuffdiff software (Trapnell et al., 2010). The expression levels of gene were computed by summing the FPKMs of transcripts in each group. Differential expression levels were determined by Cuffdiff using a model based on the negative binomial distribution model. Transcripts with a corrected *P*-value < 0.05 were considered significantly differentially expressed.

### GO and KEGG Enrichment Analyses

Analyses of Gene Ontology (GO) enrichment and Kyoto Encyclopedia of Genes and Genomes (KEGG) signaling pathway (www.kegg.jp/kegg/kegg1.html) were performed to investigate the roles of differentially expressed lncRNA and mRNAs by the GOseq R package and KOBAS v2.0 software (Xie et al., 2011), respectively. Corrected *P*-value < 0.05 was considered significantly enriched.

### Association Analysis Between lncRNA and CPID

To deepen our understanding of the relationship between lncRNAs, these target genes and CPID, three criteria were performed to screen lncRNA and the target mRNA pairs. Firstly, the predicted lncRNAs and their target genes were both significantly differentially expressed in IR vs. IC and/or IS vs. IC groups through *cis* and *trans* roles. Secondly, the CPID-related functional mRNA set was searched and downloaded from the GeneCard database: Genes Associated with Diseases (http://www.genecards.org/cgi-bin/listdiseasecards.pl), the CPID-associated keywords were used to screen gene pairs, such as *C. perfringens*, diarrhea, inflammation, immune, infection, and inflammatory bowel disease (IBD). Lastly, compared with functional mRNA set, only the matched gene pairs were selected and considered as potential CPID-related gene pairs. The screening process was outlined in Figure S1. Function analyses were also performed to investigate the functions of these lncRNAs and target genes in regulating CPID. Furthermore, these gene pairs were processed to predict the potential regulatory relationship between lncRNAs and immune-related genes, which were utilized to further explore the immune function of lncRNAs in regulating CPID.

### Quantitative PCR Validation

To validate RNA-seq data, the expression levels of 12 genes from multiple groups were quantified by quantitative PCR (qPCR) using 2^−ΔΔCt^ value methods. Ileum total RNA used for RNA-seq was performed to synthesize cDNA using reverse transcriptase Kit (TaKaRa, Dalian, China). Specific primers of these genes were designed using NCBI database, porcine *GAPDH* gene was designed as an endogenous control (Table S1). The qPCR reactions were performed in 20 μL system involved in 9.5 μL 2 × SYBR Green Realtime PCR Master Mix (TaKaRa, Dalian), 1 μL of forward and reverse primers, 1 μL cDNA and 7.5 μL RNase free ddH_2_O using LightCycler 480 II Real-Time PCR System. The cycling conditions included an initial denaturation (95°C for 3 min), followed by 30 cycles (95°C for 15 s; 60 ± 1°C for 15 s; 72°C for 20 s). Three independent biological replicates were performed in triplicate.

## Results

### Effect of *C. perfringens* Type C Infection on Fecal Bacterial Shedding

**Figure 1:**
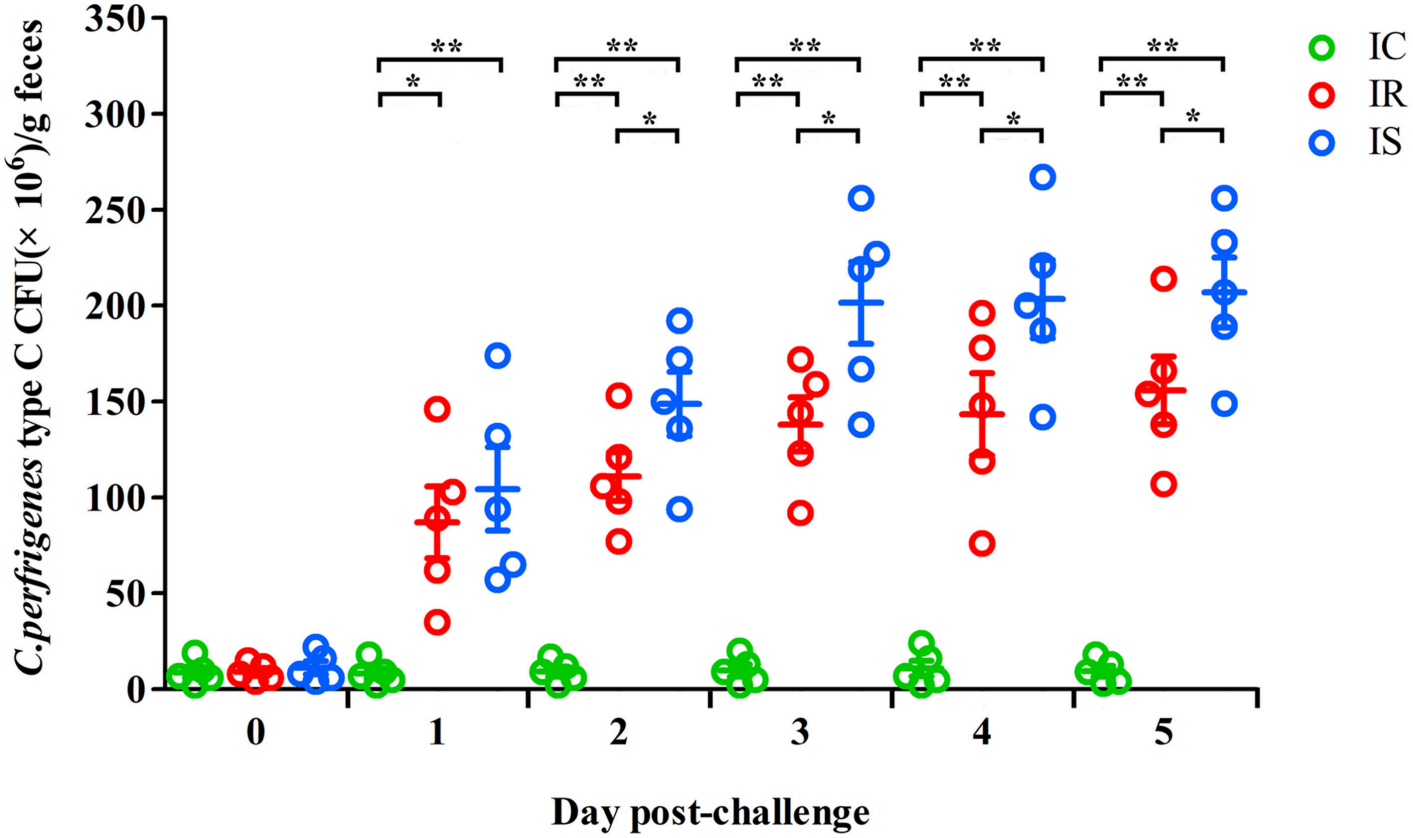
The fecal shedding levels of piglets in IR, IS, and IC groups from 0 to 5 days post challenge (dpc). Fecal shedding CFUs were determined by plate count method. The horizontal line represents the mean. Green circle represents results of IC; Red circle represents results of IR; Blue circle represents results of IS. An asterisk denotes a significant difference (^*^P < 0.05; ^**^P < 0.01).

To explore the effect of *C. perfringens* type C infection on the fecal bacterial shedding, the numbers of fecal bacterial shedding of piglets from the IR, IS and IC groups were measured on 1–5dpc. The fecal *C. perfringens* type C shedding in the IR and IS groups were increasing over time. The mean values of *C. perfringens* type C CFUs in the IR group were significantly lower for the IR group than those in the IS group, which were both significantly higher than those in the IC group (*P* < 0.01) (Figure 1). The results suggested that *C. perfringens* type C infection could increase the numbers of fecal bacterial shedding, the hosts with more severe diarrhea shed more bacteria in their feces.

### Summarized RNA Sequencing Data

To study the expression profiles of lncRNA and mRNA of piglets, RNA sequencing was performed on ileum tissues of 15 piglets from the IR, IS, and IC groups. Through sequencing, each tissue sample generated a total of 90 to 130 million raw reads, after discarding reads with poly-N >10%, adapters, low quality reads, and/or any other contaminants, ~83–125 million clear reads were obtained. The percentage of clean reads in each library ranged from 88.95 to 96.36%, and an average of 92.97% clean reads passed initial quality thresholds. Upon aligning to the pig reference genome (*Sus scrofa* 10.2), ~59–89 million clean reads (average 65.89% clean reads) were mapped (for details of sequencing results see Table S2).

### Identification of lncRNA and mRNA

**Figure 2:**
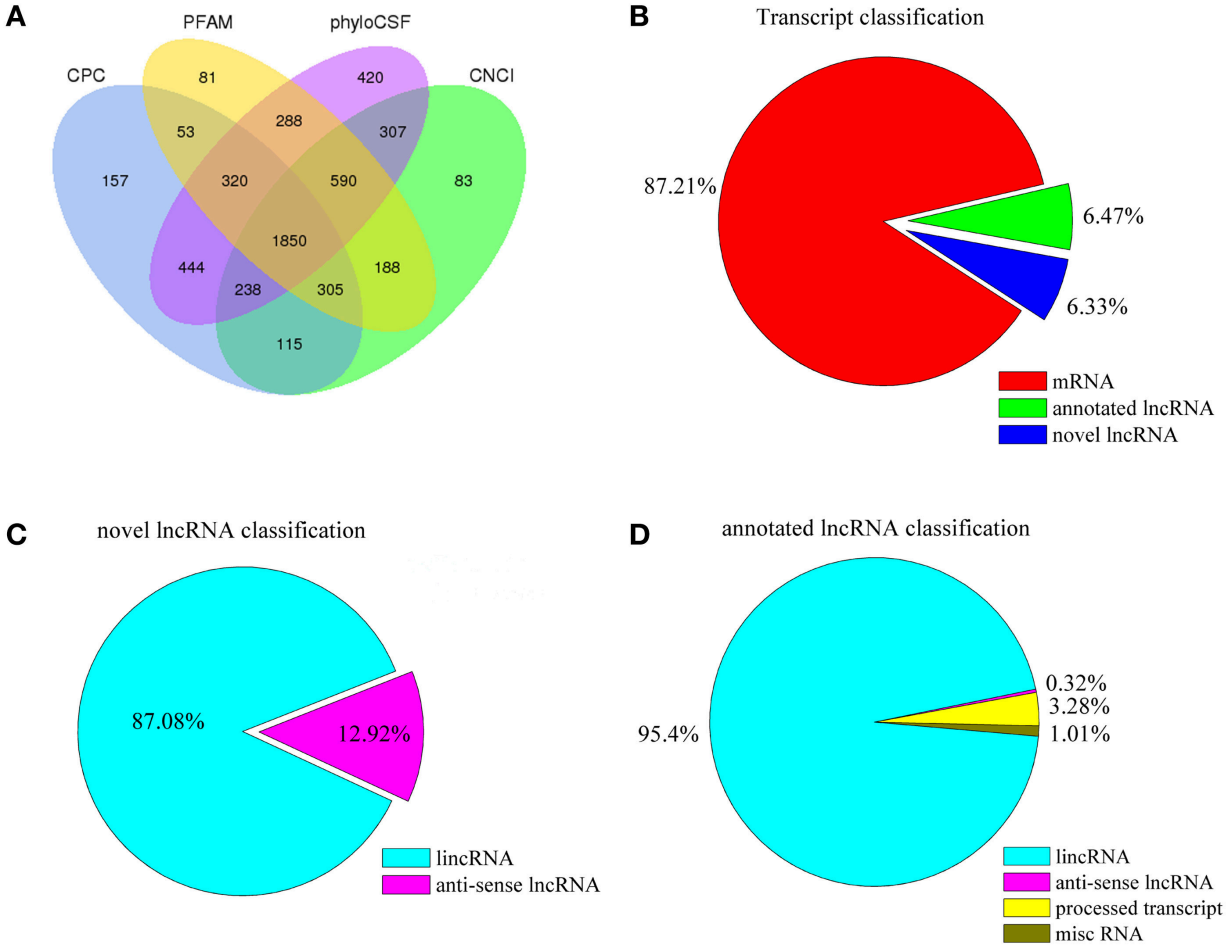
**(A)** Identification of 1,850 putative lncRNAs without protein-coding potential evaluated by CPC, PFAM, phyloCSF, and CNCI. **(B)** Classification of lncRNAs, **(C)** novel lncRNAs, **(D)** annotated lncRNAs, and mRNAs identified in piglet ileums by RNA-seq.

To identify the high-confidence lncRNAs, we first reconstructed and assembled 163,927 non-redundant transcripts by two assemblers: Cufflinks and Scripture. Then, a series of highly stringent bioinformatics filtering pipelines was applied to screen putative lncRNAs (Figure S2). Since transcripts involved immature mRNA fragments, four bioinformatic tools, namely CPC, PFAM, phyloCSF, and CNCI, were used to assess the coding potential of transcripts, and only transcripts that were simultaneously shared by none of four tools were designated as putative lncRNA. This strategy yielded a “high-confidence” set of 1,850 putative novel lncRNAs (Figure 2A, Figure S3), corresponding to 1,393 lncRNA genes and representing 1,611 lincRNAs (87.08%) and 239 anti-sense lncRNAs (12.92%) (Figure 2C). In addition, 1,890 annotated lncRNAs were identified, corresponding to 1,650 lncRNA genes and representing 1,803 lincRNAs (95.4%), 62 processed transcripts (3.28%), 19 miscRNAs (1%), and 6 anti-sense lncRNAs (0.32%) (Figure 2D and Table S3-1). In addition, a total of 25,491 mRNAs were identified (Figure 2B and Table S3-2). We henceforth referred these sets as the “pig intestinal transcriptome,” and all subsequent analyses were based on these transcripts.

### Genomic Feature Analyses of lncRNA and mRNA

**Figure 3:**
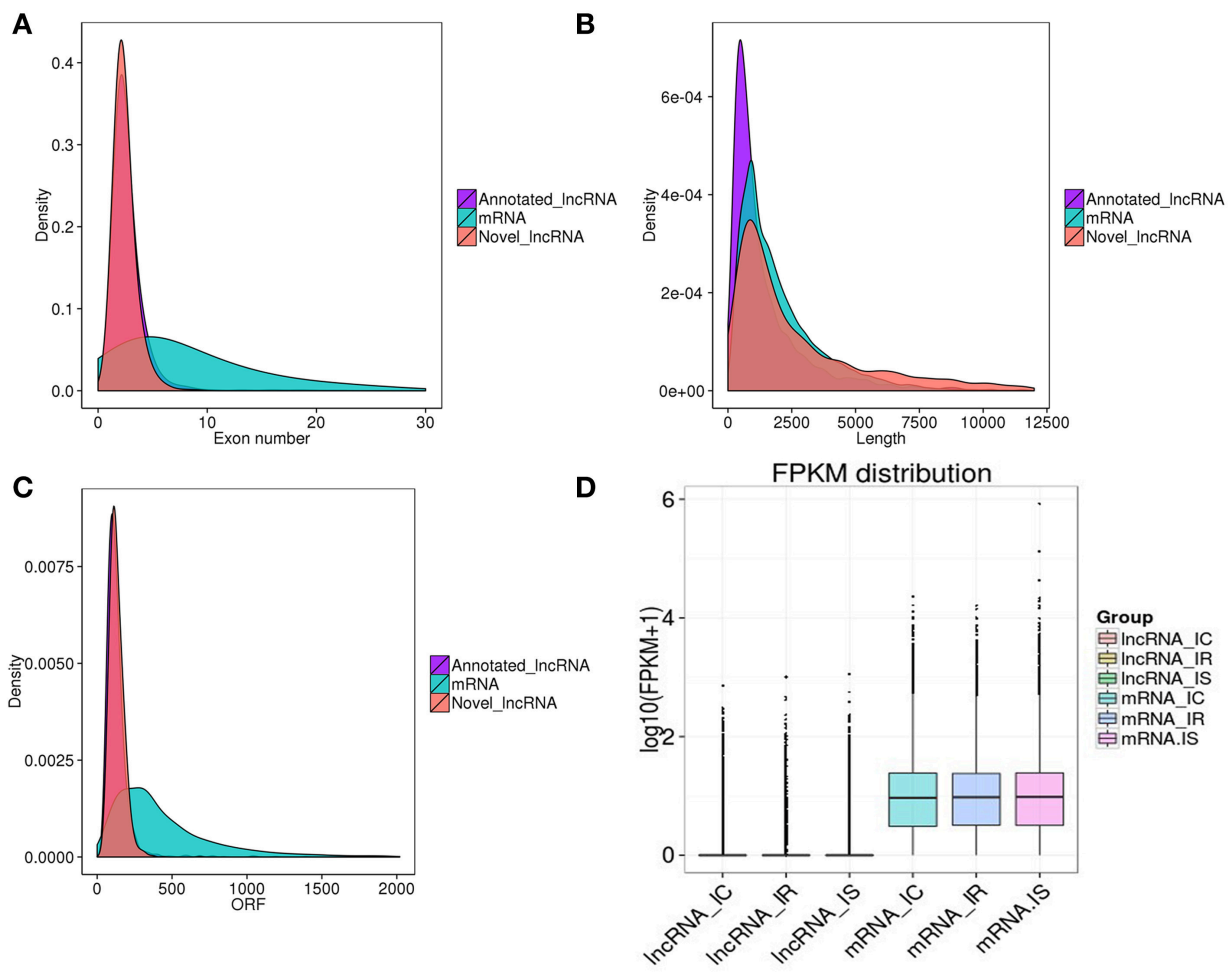
Genomic characteristic of lncRNAs and mRNAs in piglet ileums. **(A)** Exon number distribution of lncRNAs and mRNAs; **(B)** Length distribution of 1,850 annotated lncRNAs (purple), 25,491 mRNAs (green), and 1,890 putative novel lncRNAs (red); **(C)** ORF length distribution of lncRNAs and mRNAs; **(D)** Box plots showing the expression levels (log 10 FPKM) of lncRNAs and mRNAs in IR, IS, and IC groups.

To investigate the genomic features of these predicted transcripts, we characterized the exon number, transcript length, open reading frame (ORF) length and sequence conservation of lncRNAs and mRNAs. In agreement with other lncRNAs, the predicted lncRNAs in this study were shorter in length and had less exons, ORF and lower conservation than mRNAs (Figures 3A–C). Specifically, the 1,890 annotated lncRNAs had an average of 1,546bp lengths, 2.77 exons and 129bp ORF, being shorter than the 1,850 novel lncRNAs, which had an average of 3,496bp lengths, 2.44 exons and 131bp ORF (Table S4). Interestingly, we also found that lncRNAs in piglet ileums were not only longer in length than those in pig endometrium (1,454bp on average) (Wang et al., 2016), skeletal muscle (1,043bp on average) (Zhao et al., 2015), testis (1,240bp on average) (Ran et al., 2016) and thyroid gland (2,337bp on average) (Shen et al., 2016), but also longer than those in human (1kb on average), mouse (550bp on average), and zebrafish (1,113bp on average). In addition, the predicted lncRNAs of pig contained the fewer numbers of exon than those of human (2.9 exon on average), mouse (3.7 exon on average) and zebrafish (2.8 exon on average) (Siepel et al., 2005; Cabili et al., 2011; Pauli et al., 2012).

### Differential Expression Analyses of lncRNA and mRNA

To identify the expression levels of lncRNAs and mRNAs, Cuffdiff software was first applied to screen differentially expressed lncRNAs and mRNAs by FPKM. Consistent with other species, the expression levels of lncRNAs were much lower than those of mRNAs (Figure 3D). With the false discovery rate (FDR) set at 4% and corrected *P*-value of < 0.05, a total of 480 lncRNAs and 3,669 mRNAs were significantly differentially expressed among the pairwise comparisons (IR vs. IC, IS vs. IC, and IR vs. IS) (Table S5). The 480 dysregulated lncRNAs represented 237 novel lncRNAs and 243 annotated lncRNAs, corresponding to 478 lncRNA genes (Table S5-1), a total of 359, 16 and 419 lncRNAs were specifically expressed in IR vs. IC, IS vs. IC, and IR vs. IS groups, respectively (Table 1). Meanwhile, the 3,669 dysregulated mRNAs corresponded to 3,663 mRNA genes (Table S5-2), and 2,588, 126 and 3,283 mRNAs were found to be differentially expressed in IR vs. IC, IS vs. IC, and IR vs. IS groups (Table 1), respectively.

**Table 1:** Number of differentially expressed lncRNAs and mRNAs in each comparison.

**Type**	**Groups**	**IR vs. IC**	**IR vs. IS**	**IS vs. IC**	**Total genes**
lncRNA	Up-regulated	84	6	108	480
	Down-regulated	275	10	311	
	Sum	359	16	419	
mRNA	Up-regulated	1,419	41	1,825	3,669
	Down-regulated	1,169	85	1,458	
	Sum	2,588	126	3,283	

**Figure 4:**
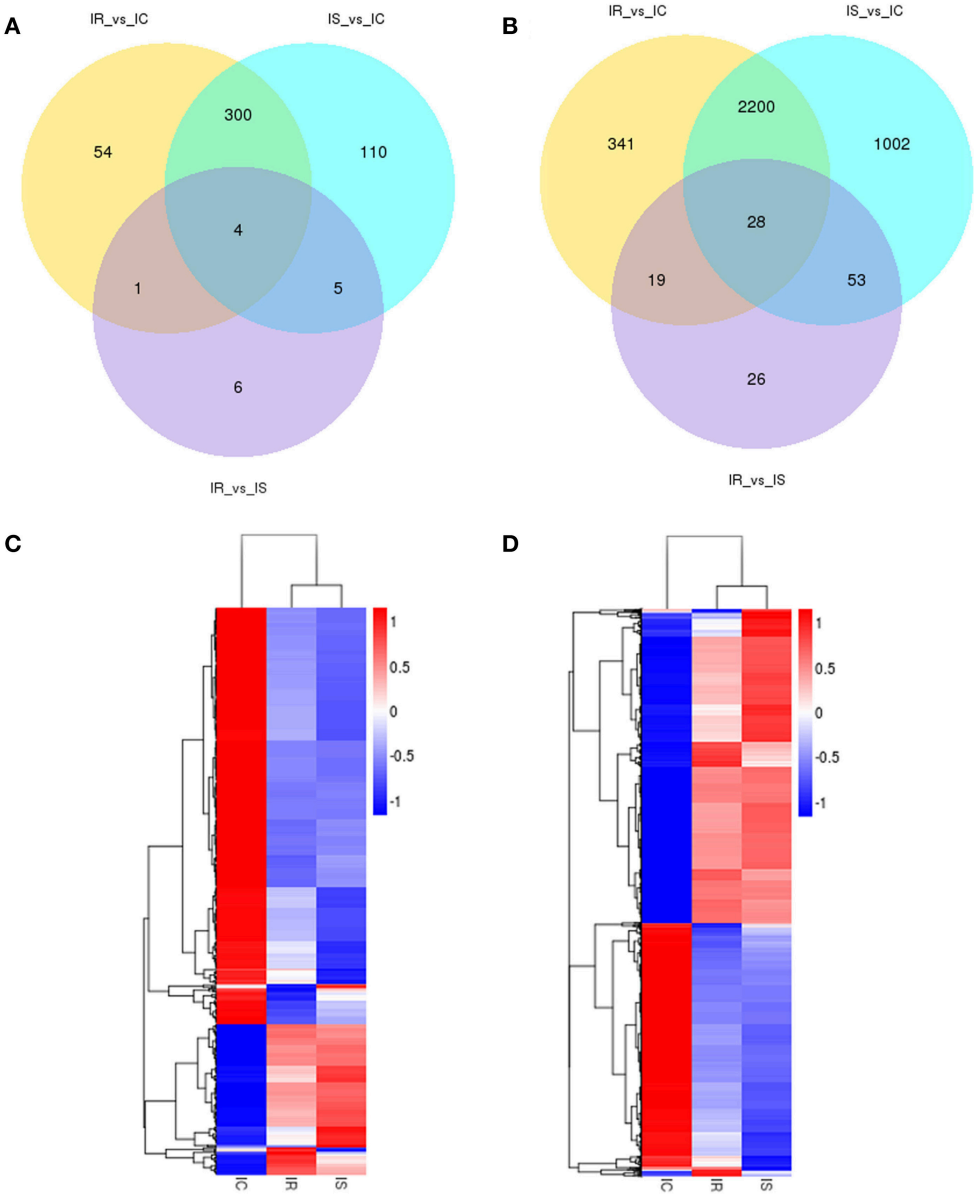
Gene expression number and profiling analyses of differentially expressed lncRNAs and mRNAs among IR, IS, and IC groups after *C. perfringens* type C infection. Venn diagram of differentially expressed lncRNAs **(A)** and mRNAs **(B)** in three comparison groups. Cluster analyses of differentially expressed lncRNAs **(C)** and mRNAs **(D)** of 15 piglets infected by *C. perfringens* type C by hierarchical heat map. Data are expressed as FPKM. Red, relatively higher expression level; Blue, relatively lower expression level.

Venn diagraming was performed to describe overlaps among the differentially expressed lncRNAs and mRNAs from pairwise comparisons. In total, 54, 110 and 6 stage-specific lncRNAs were differentially expressed in IR vs. IC, IS vs. IC, and IR vs. IS groups, respectively. Four dysregulated lncRNAs (ENSSSCT00000032859, ENSSSCT00000018610, LNC_001066 and LNC_001186) were shared among the IR, IS, and IC groups (Figure 4A). Moreover, a set of 341, 1,002, and 26 dysregulated mRNAs were found to be specifically expressed in the IR vs. IC, IS vs. IC, and IR vs. IS groups, respectively, and 28 dysregulated mRNAs were identified as the transcripts shared among IR, IS, and IC groups (Figure 4B). Taken together, the set of lncRNAs and mRNAs suggested the existence of a global coordination for regulatory responses in piglet intestine immune response to *C. perfringens* type C infection. In addition, systematic cluster analyses of differentially expressed lncRNA and mRNAs among IR, IS, and IC groups were revealed by heat map. The lncRNAs (Figure 4C) and mRNAs (Figure 4D) of IR and IS showed similar expression patterns being clustered together.

**Figure 5:**
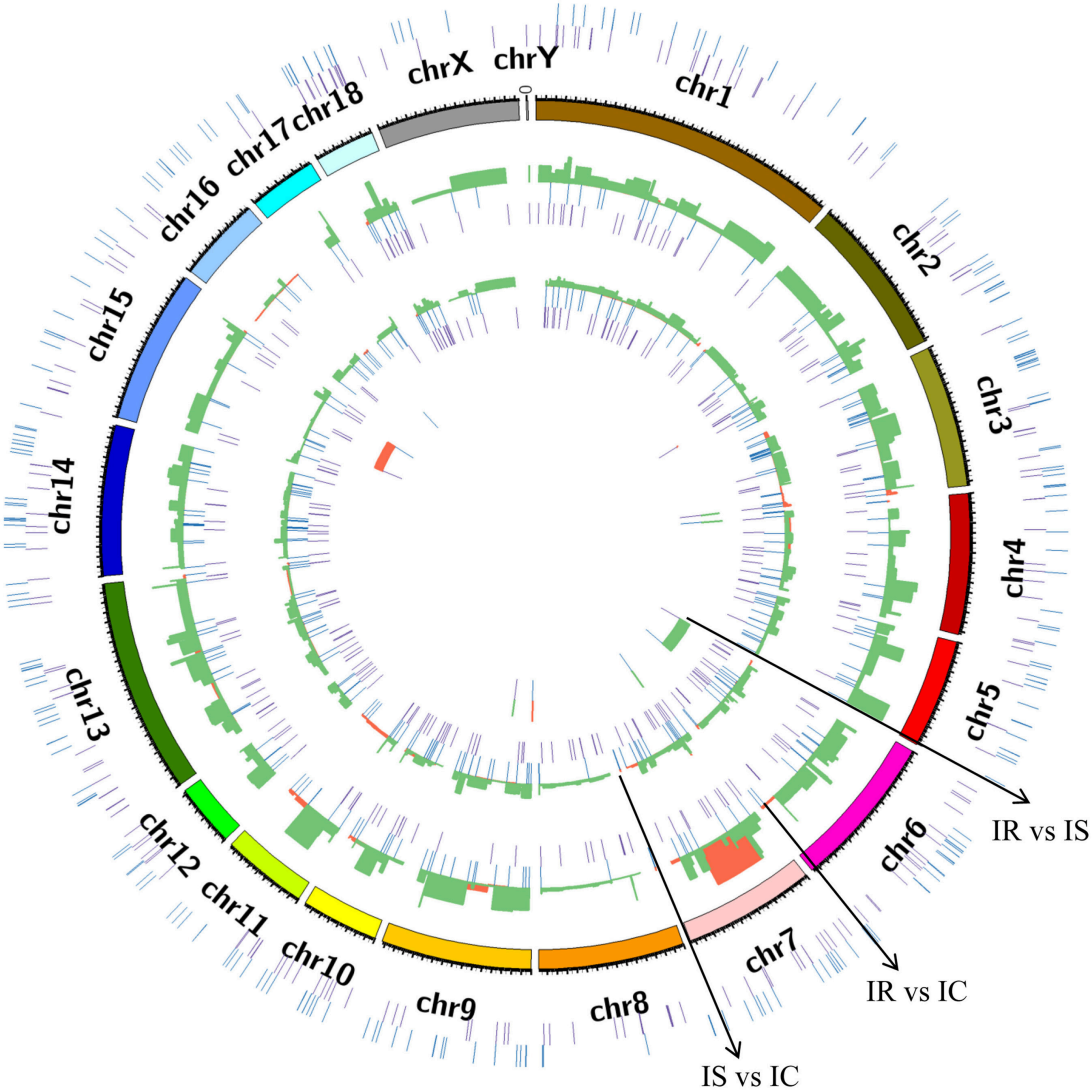
Circular chromosome distribution of lncRNA identified in piglet ileums across the reference genome and different comparison groups. The outermost circle displays the sum of novel and annotated lncRNAs identified in three comparison groups. The second circle displays pig chromosomes scale. The remaining three circles display the novel and annotated lncRNAs in IR vs. IC, IS vs. IC, and IR vs. IS, respectively. Purple: novel lncRNA; Blue: annotated lncRNA; Red: the up-regulated lncRNA; Green: the down-regulated lncRNA, the red and green were plotted by the log2 (fold change) among IR vs. IC, IS vs. IC, and IR vs. IS groups.

To explore the genomic distribution of differentially expressed lncRNAs, a circular chromosomal distribution was generated to display the differential expression patterns of novel and annotated lncRNAs in IR vs. IC, IS vs. IC, and IR vs. IS groups (Figure 5). The distribution and expression patterns of dysregulated lncRNAs were similarly between the IR vs. IC and IS vs. IC comparisons, and the dysregulated lncRNAs mainly represented the down-regulated transcripts, evenly distributed among nearly all chromosomes. In contrast, the majority of up-regulated lncRNAs were distributed among chromosomes 7, 9, and 11 for the IR vs. IC comparison, and chromosomes 3, 4, 7, and 11 for the IS vs. IC comparison. For the IR vs. IS comparative expressions, the up- and down-regulated lncRNAs were mainly distributed in chromosomes 15 and 6, respectively.

### Function Enrichment Analysis of Differentially Expressed lncRNAs

To gain insight into the potential function of the infection-related differentially expressed lncRNAs, GO and KEGG enrichment analyses of lncRNAs in *cis* and *trans* regulatory roles were performed. We first predicted the potential target genes located in the 10-kb (upstream and downstream) regions of lncRNAs. A total of 1,728 lncRNAs were found to transcribe close to 2,104 mRNAs, corresponding to 2,672 lncRNA: coding gene pairs (Table S6-1). Through no significantly enriched GO terms were identified in the IR vs. IC and IS vs. IC groups (corrected *P*-value > 0.05, Tables S7, S8), target genes of these lncRNAs were also found to enrich in some immune-related signaling pathways, such as the B cell receptor signaling pathway, chemokine signaling pathway, NF-κB signaling pathway, NOD-like receptor signaling pathway and T cell receptor signaling pathway. It was notably that up-regulated lncRNAs in the IS vs. IC group were significantly enriched in the ABC transporters pathway (corrected *P*-value = 0.0178). GO survey indicated that the *cis* lncRNA target genes in 13 GO terms were significantly enriched in IR vs. IS group (corrected *P*-value < 0.05, Table S7-1), these enriched terms were mostly associated with the receptor activity and detection of stimulus, such as olfactory receptor activity, G protein-coupled receptor activity, transmembrane signaling receptor activity, detection of chemical stimulus involved in sensory perception and smell. Notably, in IR vs. IS group, the target genes of up-regulated lncRNAs were mainly enriched in two pathways, olfactory transduction (corrected *P*-value = 0.0071) and nitrogen metabolism (corrected *P*-value = 0.0362), while the target genes of down-regulated lncRNAs were enriched in one pathway (protein processing in endoplasmic reticulum; corrected *P*-value = 0.0071), these enriched GO classes and KEGG pathways mainly revealed two genes, *CPS1* and *OR6A2*, hinting the key roles of these two genes. Importantly, these pathways enriched in the IR vs. IS group might be significantly associated with the resistance and susceptibility of host response to *C. perfringens* infection (Table S8-1).

We further predicted potential target genes of lncRNAs in *trans* regulation based on Pearson's correlation coefficients (|*r*| > 0.95). A total of 17,895 interaction relationships (17,654 positive and 241 negative correlations) were identified, representing 781 dysregulated lncRNAs and 2,949 target genes (Table S6-2). Furthermore, the *trans* target genes of lncRNAs were significantly enriched in 198, 3 and 187 GO terms for IR vs. IC, IR vs. IS, and IS vs. IC comparisons, respectively (corrected *P*-value < 0.05, Table S7-2). Remarkably, the 3 enriched GO terms in the IR vs. IS group included cytokine production, regulation of immune response and positive regulation of immune system process, and four immune-related target genes, *IRAK3, LCP2, TLR8*, and *CD84*, were annotated in them (corrected *P*-value < 0.05, Table S7-2). Similar to lncRNAs in *cis* role, the major categories of target genes of lncRNAs in *trans* roles from IR vs. IC, IS vs. IC, and IR vs. IS groups mainly included olfactory transduction, transcriptional misregulation in cancer (*IL-8, CD86*, and *MMP-3*), osteoclast differentiation (*TGFBR1, IL-1A*, and *TNFRSF11A*), immune- and inflammation-related pathways (inflammation signaling-*JUK* and *NLRP3*; cytokine-*CCL5* and *IL-1*; immune receptor- *NOD1, TLR3*, and *IRAK3*) (Table S8-2).

### Function Enrichment Analyses of Differentially Expressed mRNAs

Gene set enrichment analyses of differentially expressed mRNAs of piglets revealed that a total of 145, 23, and 257 highly enriched GO terms were derived from IR vs. IC, IR vs. IS, and IS vs. IC groups, respectively (corrected *P*-value < 0.05, Table S7-3). The most enriched GO terms in three comparison groups included the broad functional clustering, especially various inflammatory-related functions (cytokine, leukocyte activation, chemotaxis, and lymphocyte differentiation) and cell metabolic process (catalytic activity, hydrolase, hemopoiesis). In the KEGG analysis, the most significant pathways of mRNAs included cGMP-PKG signaling pathway (*INOS*), ABC transporters (*ABCB1*) and PPAR signaling pathway (*PPAR-*α). It's worth noting that the dysregulated mRNAs in the IR vs. IS group significantly activated some immune-related GO functions, these terms included mucosal immune response, defense response, antioxidant activity, sulfiredoxin activity (corrected *P*-value < 0.05, Table S7-3). Furthermore, the enrichment pathways of up-regulated mRNAs in the IR vs. IS group were significantly enriched in only two immune-related pathways: chemokine signaling pathway (corrected *P*-value = 0.0161) and Toll-like receptor signaling pathway (corrected *P*-value = 0.0218), in which, 6 immune-related mRNAs (*CXCL9, CXCL10, CCL17, CCR5*, and *TLR8*) were found to annotate in these pathways. Other pathways of dysregulated mRNAs included glycosphingolipid biosynthesis and protein processing in endoplasmic reticulum (corrected *P*-value = 0.0357) (Table S8-3). In general, the significant functions of mRNAs that changed during infection might be linked to the induction of this gene in the regulation process of host immune response to *C. perfringens* type C infection.

### Association Analysis Between lncRNA, Target Genes and CPID

**Figure 6:**
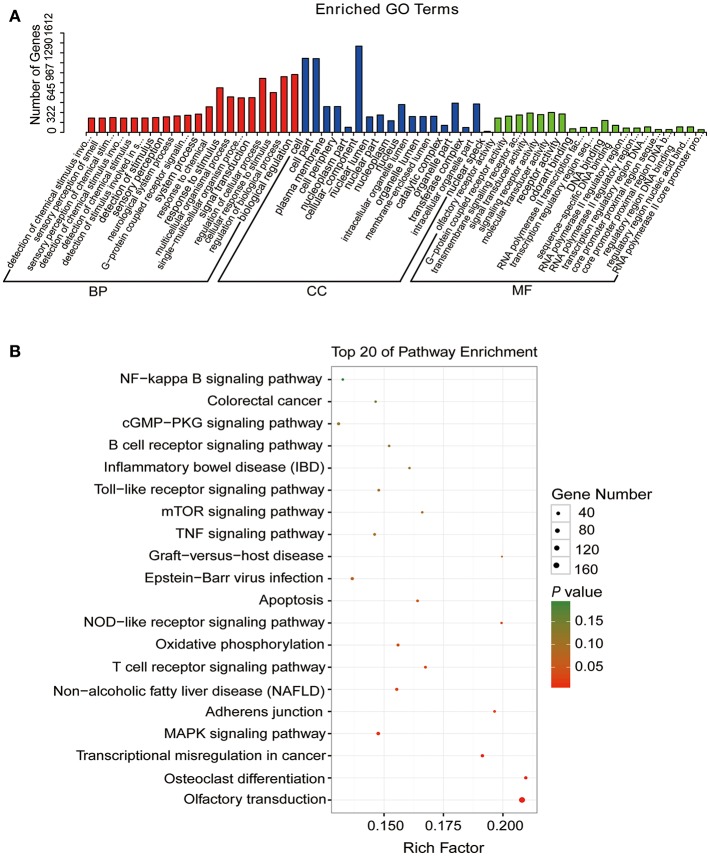
The functional enrichment analyses of target genes of the disease-related lncRNAs. **(A)** Gene ontology (GO) function annotation of target genes of the disease-related lncRNAs. The X-axis indicates the gene numbers and the Y-axis indicates the detailed terms. **(B)** Kyoto encyclopedia of genes and genomes (KEGG) pathways of target genes of the disease-related lncRNAs. The X-axis indicates the gene ratio and the Y-axis indicates the name of KEGG pathway. The size of dots indicates the numbers of target genes, and the color of dots indicates *P*-value (Fisher's Exact Test).

To study the association between lncRNAs and CPID, we had implemented three criteria to screen for the most likely lncRNAs involved in the regulation of CPID. A total of 212 lncRNAs, corresponding to 505 target genes were filtered (Table S9). GO analysis of these candidate gene pairs showed that these lncRNA target genes were mainly involved in cellular response to stimulus, regulation of cellular and biological processes, signal transduction and activity, cellular component, and G protein-coupled receptor activity and so on (Figure 6A). The significantly enriched KEGG pathways involved MAPK signaling pathway, T cell receptor signaling pathway, NOD-like receptor signaling pathway, apoptosis, adhesion junction, Toll-like receptor signaling pathway and NF-κB signaling pathway (Figure 6B). The potential candidate gene pairs could regulate the process of *C. perfringens* type C infection through these functions and pathways.

**Figure 7:**
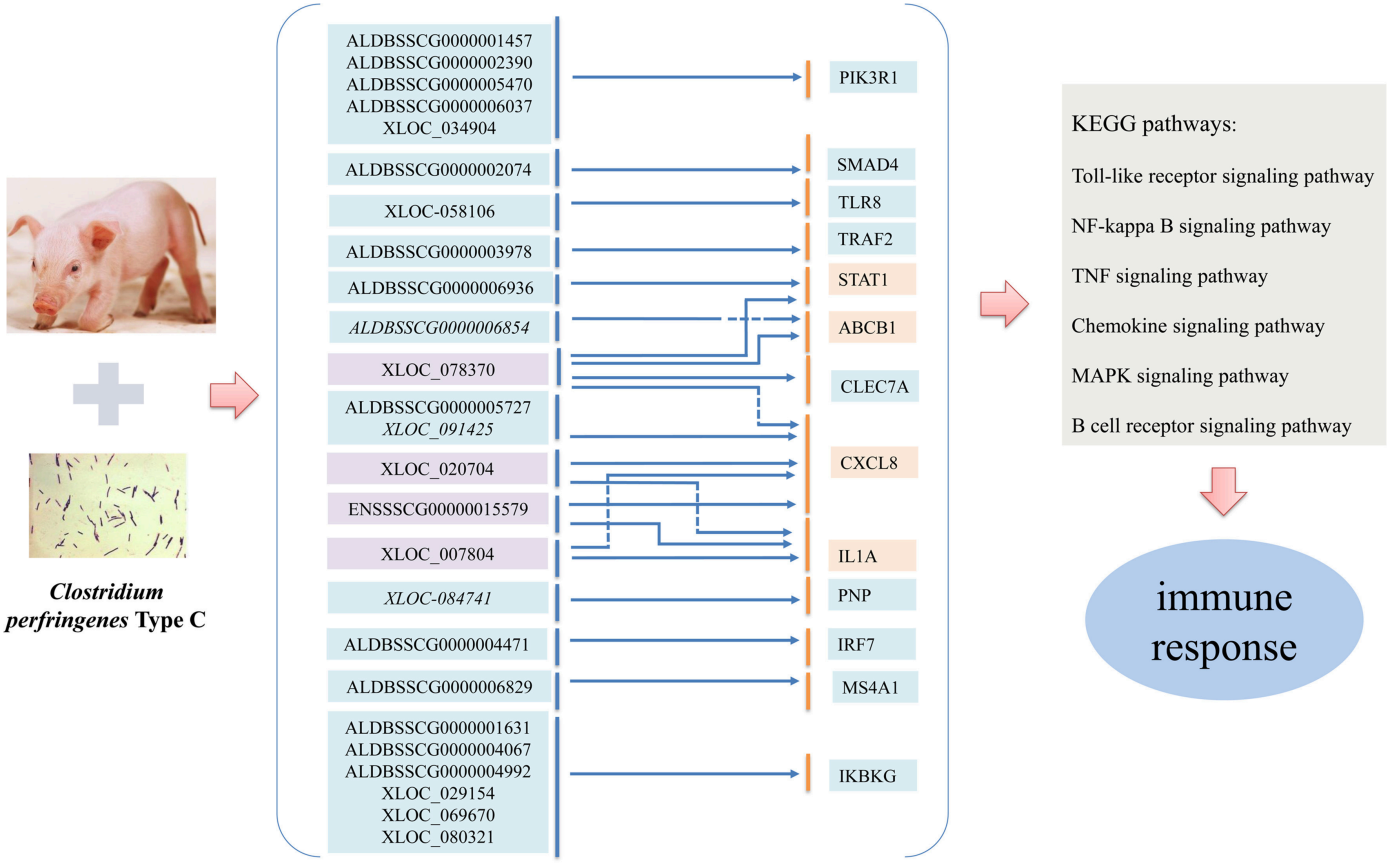
Overview of the regulatory relationship between the differentially expressed lncRNAs and their immune-related target genes involved in CPID. Italics and non-italics fonts of lncRNA indicate the *cis* and *trans* regulatory functions of lncRNAs, respectively.

To study the immune regulatory functions of lncRNAs, the potential relationships of differentially expressed lncRNAs and immune-related genes were predicted. Finally, a total of 25 dysregulated lncRNAs targeting 13 immune-related genes passed the filter (Figure 7) and were used to perform the enrichment analyses. Several of the lncRNAs had multiple immune-related target genes and a given immune target gene could be regulated by several lncRNAs. In particular, *ABCB1*, a significantly dysregulated immune-related gene, was found to be regulated by XLOC_078370 in *cis* and by ALDBSSCG0000006854 in *trans*, respectively. Simultaneously, XLOC_078370, together with ALDBSSCG0000005727, ENSSSCG00000015579, XLOC_007804, and XLOC_020704, could co-regulate target gene *CXCL8* in *trans*; *CXCL8* was also regulated by XLOC_091425 in *cis*. In addition, XLOC_078370 could also target *STAT1* and *CLEC7A* genes, both of which were significantly differentially expressed in IR, IS and IC groups.

### Validation of Expression Levels of Genes Detected in RNA-Seq

**Figure 8:**
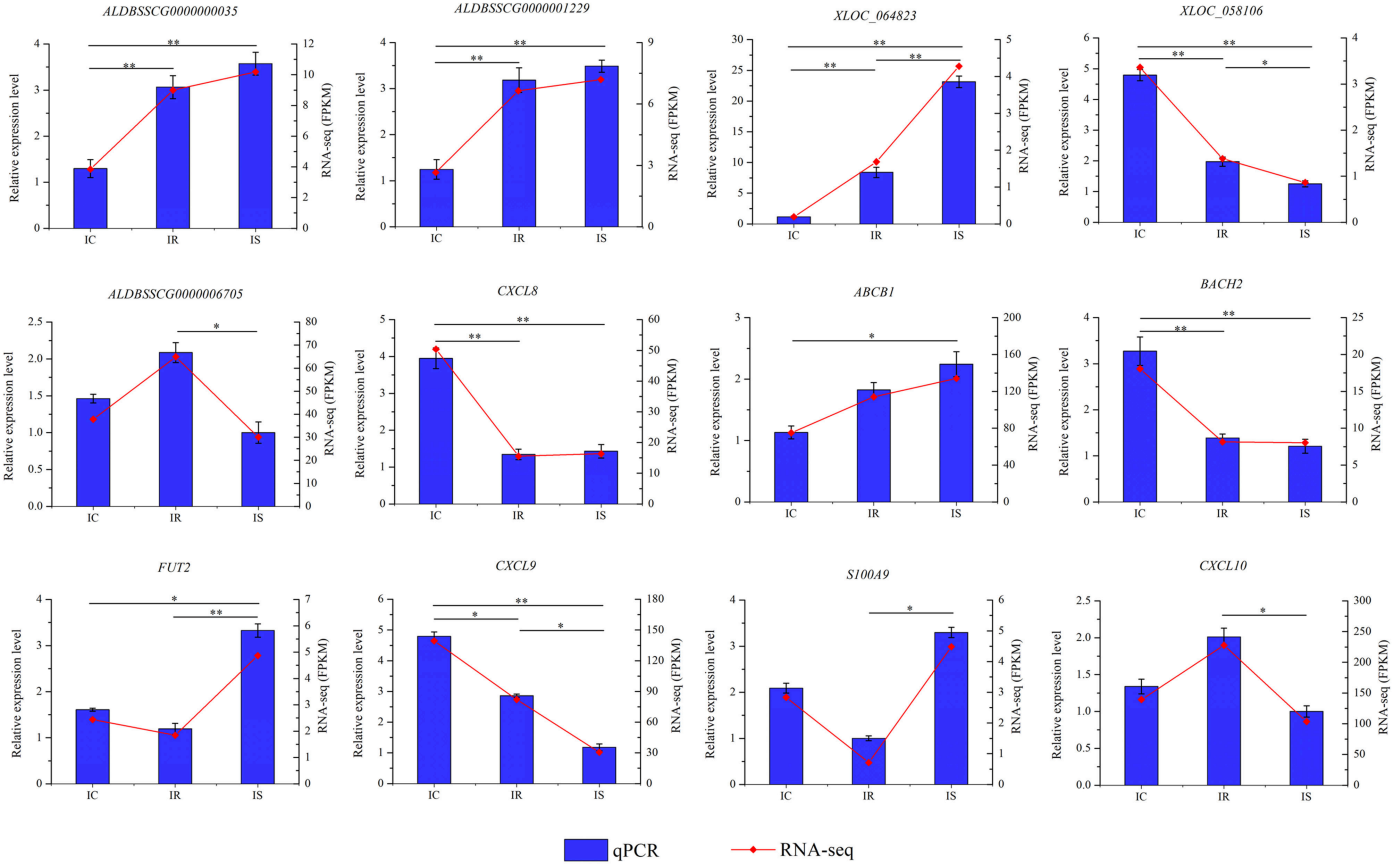
Validation of RNA-seq results by quantitative reverse transcription polymerase chain reaction (qPCR). Porcine *GAPDH* gene is used as the endogenous control. Expression was quantified using the comparative cycle threshold (2^−ΔΔCt^) value method. The data were expressed as the mean ± SEM). An asterisk denotes a significant difference (^*^P < 0.05; ^**^P < 0.01).

To validate the RNA-seq result and detect expression levels of differentially expressed genes in three comparison groups, a total of 12 dysregulated genes were subjected to qPCR detection, including 5 lncRNAs (XLOC_064823, XLOC_058106, ALDBSSCG0000000035, ALDBSSCG0000001229, and ALDBSSCG0000006705) and 7 mRNAs (*CXCL8, CXCL9, ABCB1, BACH2, FUT2, CXCL10*, and *S100A9*). The qPCR results of all 12 genes were perfectly consistent with the RNA-seq data (Figure 8), suggesting the high reliability and accuracy of RNA-seq.

## Discussion

*C. perfringens* has been described for decades years, it is generally recognized as one of the most widespread potential bacterial pathogens in nature as well as in the gastrointestinal tracts of most animal species. However, due to a paucity of studies on mechanisms of host response to *C. perfringens* type C infection, no adequate treatment or cure is yet available for the manifested diseases. In general, the complexity of mechanisms underlying human and animal infections by pathogenic bacteria is appreciated, and the related research has begun to focus on the coordinated posttranscriptional regulatory functions of host genes, such as microRNAs seem to have a relationship with chicken necrotic enteritis disease caused by *C. perfringens* infection (Hong et al., 2014), lncRNAs have been found to be implicated in regulating the bacterial infection diseases in piglets and mice (Zhao, 2016; Wu, 2018). However, no studies to date had yet performed a comprehensive assessment of lncRNA in regulation of piglet defense response against *C. perfringens* type C infection, therefore, in our study, the primary goal was to identify *C. perfringens* type C infection-related lncRNAs and mRNAs as well as the potential functions in the 7-day-old piglets from IR, IS and IC groups. According to the results, these lncRNAs and mRNAs may constitute potential candidates for future application of preventing and treating CPID.

The degree of bacterial shedding in feces is an important parameter that affects herd disease spread, as well as a measure of host's immunologic control of bacteria replication. In this study, the numbers of fecal bacterial shedding of piglets were significantly higher in IS than in IR and IC, and that in IR was significantly higher than that in IC. These findings were in accordance with the feces' consistency of three groups, suggesting that *C. perfringens* infection could induce different bacterial shedding levels of piglets that might be associated with the different immune abilities of piglets. Therefore, screening pigs with reduced *C. perfringens* numbers, may decrease environmental contamination and lower pathogen transmission to other animals and humans. Certainly, the piglets with low shedding level of feces need to be further investigated.

Recently, although a total of 12,107 pig lncRNAs have been submitted to the animal lncRNA database (ALDB Release v1.0), it is still far below the total listing of 15,778 human lncRNAs (version 27) in the GENCODE database. In the present study, the identified 3,740 lncRNAs (1,850 novel lncRNAs and 1,890 annotated lncRNAs) and 25,491 mRNAs will greatly enrich the pig transcript genomic database. Non-coding and protein-coding genes are distinguished by their coding potential. In line with other organism studies (Jia et al., 2010; Esteve-Codina et al., 2011; Ni et al., 2011; Ran et al., 2016), the putative lncRNAs of our study had the fewer exons, shorter lengths, lower expression levels and less conservation than mRNAs (Figure 3), these common characteristics of lncRNAs might be associated with the pivotal functions of regulation, control and guidance. Herein, the 480 differentially expressed lncRNAs and 3,669 differentially expressed mRNAs were used to subsequently investigated into potential regulatory functions of the host in response to *C. perfringens* type C infection, though these genes still needing to be validated by experiments.

A dominant feature of differentially expressed lncRNAs and mRNAs activated in our work was that they were primarily associated with olfactory transduction and inflammasome activation, including chemokine signaling pathway and NF-κB signaling pathway. Indeed, in our study, *C. perfringens* type C infection leaded to the significantly dysregulated expressions of proinflammatory factors (*TNFAIP8, IL-1A, IL-1B, IL-4, IL-6*, and *IL-7*), interferon (*IFNE* and *IFNG*) and toll-like receptor (*TLR6, TLR8, TLR9*, and *TLR10*) of infected piglets, suggesting inflammatory response had occurred in the injury intestine portions during infection. Inflammatory response stimulated by microbial agents or necrotic cells could activate NF-κB signaling, which was required for *C. perfringens* induced reactive oxygen species (ROS) production and cytotoxicity (Li et al., 2001), thus NF-κB-regulated proinflammatory response, TLRs and interferon (IFN) regulatory responses were activated in the process of piglet immune defense response to *C. perfringens* type C infection (Kamada et al., 2013). In addition, we also found olfactory receptor family 51 subfamily E member 1 (*OR51E1*) gene was significantly decreased in the IR and IS groups, the decreased *OR51E1* were found to suppress the transcription of intestinal epithelial cell surface receptors. Similar to other study, Chen had reported the complex relationship between transduction of olfactory signaling and inflammatory response, and thought that inflammatory cytokines could contribute to olfactory neural regeneration in immune process that fighting infection or injury through the NF-κB/JNK pathway (Chen et al., 2017). Therefore, we hypothesized olfactory transduction and receptor activity were triggered by inflammatory responses and involved in the response process of piglet immune response to *C. perfringens* type C infection, which may contribute to the survive and regeneration of injured tissue.

Remarkably, the activation process of differentially expressed mRNAs mainly corresponded to several functions of antioxidant activity and sulfiredoxin activity, and pathways of the cGMP-PKG signaling pathway (*NOS2*), ABC transporters (*ABCB1*), PPAR signaling pathway (*PPARA, ANGPTL4*), and amino acid biosynthesis metabolism. The activated PPAR signaling pathway is found to limit oxygen availability by driving the energy metabolism of epithelial cells and prevent the expansion of potentially pathogenic bacteria, while it leads to an increase oxygen concentration in injured intestines during function dysfunction of host (Laura et al., 2014; Mariana et al., 2017). *NOS2*, the gene encoding inducible nitric oxide synthase (*iNOS*), is elevated in the absence of PPAR signaling pathway, and then catalyze the synthesis of NO in tissues or cells stimulated by cytokines or microbes through cGMP-PKG, PPAR, and NF-κB signaling pathways in the process of regulating cell proliferation, apoptosis and angiogenesis, while excess NO will lead to cytotoxicity, tissue damage and necrosis, and further promote the occurrence and development of inflammatory diseases (Kim et al., 2006; Vacca, 2017). In this study, the *PPARA* gene was up-expressed both in the IR and IS groups after infection, the increased expression of *PPARA* might contribute to provide a microaerophilic and anaerobic condition that benefited to growth of anaerobic pathogen *C. perfringens* type C in intestine tissues, aggravating the inflammatory and diarrhea disease of piglets. Furthermore, affected by up-expression of *PPARA*, the expressions of *NOS2* were down-regulated in the IR and IS groups, indicating that *C. perfringens* type C infection had stimulated and induced the activations of PPAR and cGMP-PKG signaling pathways, which further suppressed *NOS2* expression in infected piglets, the reduced synthesis of NO might decrease the secretion of inflammatory factors and prevent the deterioration of inflammatory disease. These results suggested that piglets might regulate the expressions of *PPARA* and *NOS2* genes to mediate immune defense responses against *C. perfringens* type C infection by cGMP-PKG and PPAR signaling pathways.

The total 212 significantly dysregulated lncRNAs corresponded to 505 unique target genes, suggested the potential relationship between the dysregulated lncRNAs, target genes and CPID. Function enrichment analyses showed that these lncRNAs and target genes primarily corresponded to several key immune-related pathways participated in regulating piglet immune responses to *C. perfringens* infection, such as MAPK signaling pathway, T cell receptor signaling pathway, NOD-like receptor signaling pathway, apoptosis, Toll-like receptor signaling pathway and NF-κB signaling pathway (Lu et al., 2009; Nagahama et al., 2013; Low et al., 2018). Specially, the 25 lncRNAs responded to 13 immune-related target genes were found to be highly associated with CPID, further exploring the regulatory relationships of them can improve the understanding of characteristics of lncRNAs and mRNAs in regulating host immunomodulation responses to *C. perfringens* infection. Due to the highly complex and diverse roles of lncRNAs and the incomplete functional prediction method, the regulatory roles of lncRNAs in host immune response to infection may be explored through mediating expressions and functions of mRNAs.

Remarkably, among the 25 lncRNAs and 13 target genes, the significantly dysregulated immune-related mRNAs (*ABCB1, STAT1, CXCL8*, and *IL1A*) are considered as the highly immune-active genes. The ATP-binding cassette subfamily B member 1 (*ABCB1*), known as multidrug resistance 1 (*MDR1* gene), was found to be targeted by ALDBSSCG0000006854 and XLOC_078370. *ABCB1* encodes the P-glycoprotein (P-gp) to protect cells from xenobiotics damage by affecting the combination between them (Dudarewicz et al., 2013). *ABCB1* is recognized as a potential target gene of high risk of IBD, colorectal cancer (CRC), Crohn's disease and ulcerative colitis (Onnie et al., 2010; Senhaji et al., 2015), *ABCB1* may regulate functions of transporter activity, ATPase activity and transmembrane movement of substances in the immune response through ABC transporters signaling pathway during infection. Thus, the overexpression of *ABCB1* gene in the IR and IS groups may be the danger signal for inflammatory intestinal disease caused by *C. perfringens* type C infection.

The signal transducer and activator of transcription factors *STAT1* was regulated by lncRNAs ALDBSSCG0000006936 and XLOC_078370. It has also been reported that activated *STAT1* mediates the expressions of proinflammatory cytokines (*CXCL10*) and interleukin production (IFN-1, IL-8, IL-10) in human IBD (Tao et al., 2013; Giles et al., 2016), down-regulated *STAT1* has been proved to reduce tumorigenesis, inflammation and gastrointestinal diseases (Ernst et al., 2008; Lamarthée et al., 2015). Therefore, low expression of *STAT1* in the IR and IS groups might reduce the cellular damage and antibacterial state by regulating cellular response to interferon and cytokine through JAK-STAT signaling pathway, which was very important for cell viability in response to different stimuli and pathogen.

*CXCL8* is an important inflammatory mediator regulated by NF-κB transcription factor and serves to modify and enhance human innate immune responses in defense against bacterial infections (Krupa et al., 2015). The expression of *CXCL8* is significantly increased in lactobacilli-treated pig intestinal epithelial cells infected by viruses, indicating that up-regulated *CXCL8* can increase protection against intestinal pathogen infections (Albarracin et al., 2017). *CXCL8* has been considered to be associated with resistance of CRC (Xiao et al., 2015), with overexpression of *CXCL8* can induce the proliferation and migration of intestinal epithelial cell (Shen et al., 2017). *CXCL8* also serves to attract T cells, dampening tissue inflammation in response to bacterial infection, such as *C. perfringens*, through NF-κB signaling pathway, chemokine signaling pathway, NOD-like receptor signaling pathway and Toll-like receptor signaling pathway. The *IL1A* gene, encoding a member of the interleukin 1 cytokine family, is a pleiotropic cytokine involving in various virus infections, immune responses and inflammatory processes (Anders, 2016). Produced by activated macrophages, *IL1A* is processed by proteolytic enzymes and releases in response to cell injury, subsequently inducing apoptosis through MAPK signaling pathway. The up-regulation of *IL1A* gene has been suggested to play an important role in preventing colon tumor in patients with IBD (Yoshikawa et al., 2017). In our study, the expressions of *CXCL8* and *IL1A* genes were significantly decreased both in the IR and IS groups, the low expressions might exert a negative impact on the piglet defense against *C. perfringens* type C infections. In general, these results strongly suggest a linkage between expressions of lncRNAs, immune-related genes and *C. perfringens* type C infection. Future studies should seek to detail mechanisms by which these lncRNAs function to regulate the target genes in pig and to confirm the findings in humans.

In summary, this study provides an overview of the expression patterns and enrichment functions of lncRNAs and mRNAs involved in the piglet immune response to *C. perfringens* type C infection. These insights into the characteristics of lncRNAs and mRNAs underlying host (piglet) immunomodulation responses to counteract the *C. perfringens* type C infection and related diseases, particularly of resistance, and provide a foundation for future studies of this pathogen infection in humans.

## Ethics Statement

This study was carried out in accordance with the recommendations of Institutional Animal Care and Use Committee (IACUC) of Gansu Research Center for Swine Production Engineering and Technology. The protocol was approved by the College of Animal Science and Technology, Gansu Agricultural University.

## Author Contributions

XH conceived and designed the study, analyzed the data, and wrote the manuscript. SG contributed to critical revising of the manuscript and final approval of the version to be published. HS and WS did the laboratory work in the expression and statistical analysis. QY, PW, and SL contributed to data analysis and interpretation. LL, SZ, and ZY participated in the analysis and interpretation of data. All the authors read and approved the manuscript.

### Conflict of Interest Statement

The authors declare that the research was conducted in the absence of any commercial or financial relationships that could be construed as a potential conflict of interest.

## Supplementary Material

Figure S1 Screening principles of association analysis between dysregulated lncRNAs and mRNAs.

Figure S2 Identification pipeline for lncRNAs. Each step is described in detail in the methods section.

Figure S3 One thousand eight hundred and fifty non-coding lncRNAs were selected by Cufflinks and Scripture.

Table S1 Primers and location of lncRNAs and mRNAs used in qPCR validation.

Table S2 Summary of RNA-seq data and reads mapped to the *Sus scrofa* reference genome.

Table S3 Catalog of lncRNAs and mRNAs identified in the 15 cDNA libraries of piglets infected by *C. perfringens* type C.

Table S4 Characteristics of lncRNAs in piglet ileums.

Table S5 List of differentially expressed lncRNAs and mRNAs detected in piglet ileums using the RNA-seq.

Table S6 Target genes prediction of significantly differentially expressed lncRNAs in *cis* and *trans*.

Table S7 GO enrichment analysis of the significantly dysregulated lncRNAs target genes and mRNAs among IR, IS, and IC groups.

Table S8 KEGG enrichment analysis of the significantly dysregulated lncRNAs target genes and mRNAs among IR, IS, and IC groups.

Table S9 Relationship between dysregulated lncRNAs and mRNAs in *cis* and *trans* involved in CPID.

## Abbreviations

CPID: *C. perfringens* infectious diseases.
