# Supplementary figures and images for: Integrative Analyses of Long Non-coding RNA and mRNA Involved in Piglet Ileum Immune Response to Clostridium perfringens Type C Infection
Source: Front Cell Infect Microbiol. 2019 Apr 30;9:130. doi: 10.3389/fcimb.2019.00130 (PMC6503642; doi:10.3389/fcimb.2019.00130)

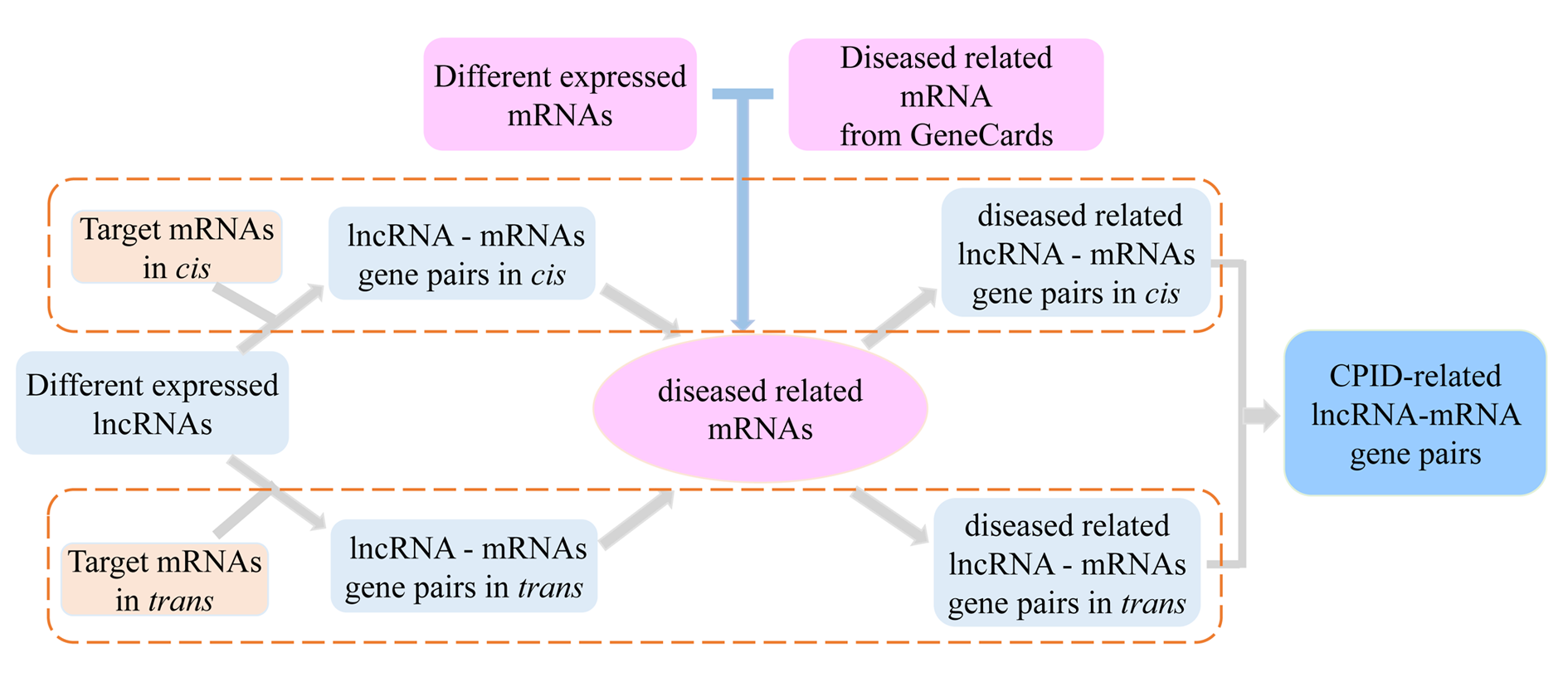

Supplement: Figure S1 — Screening principles of association analysis between dysregulated lncRNAs and mRNAs. [file Image_1.TIF]

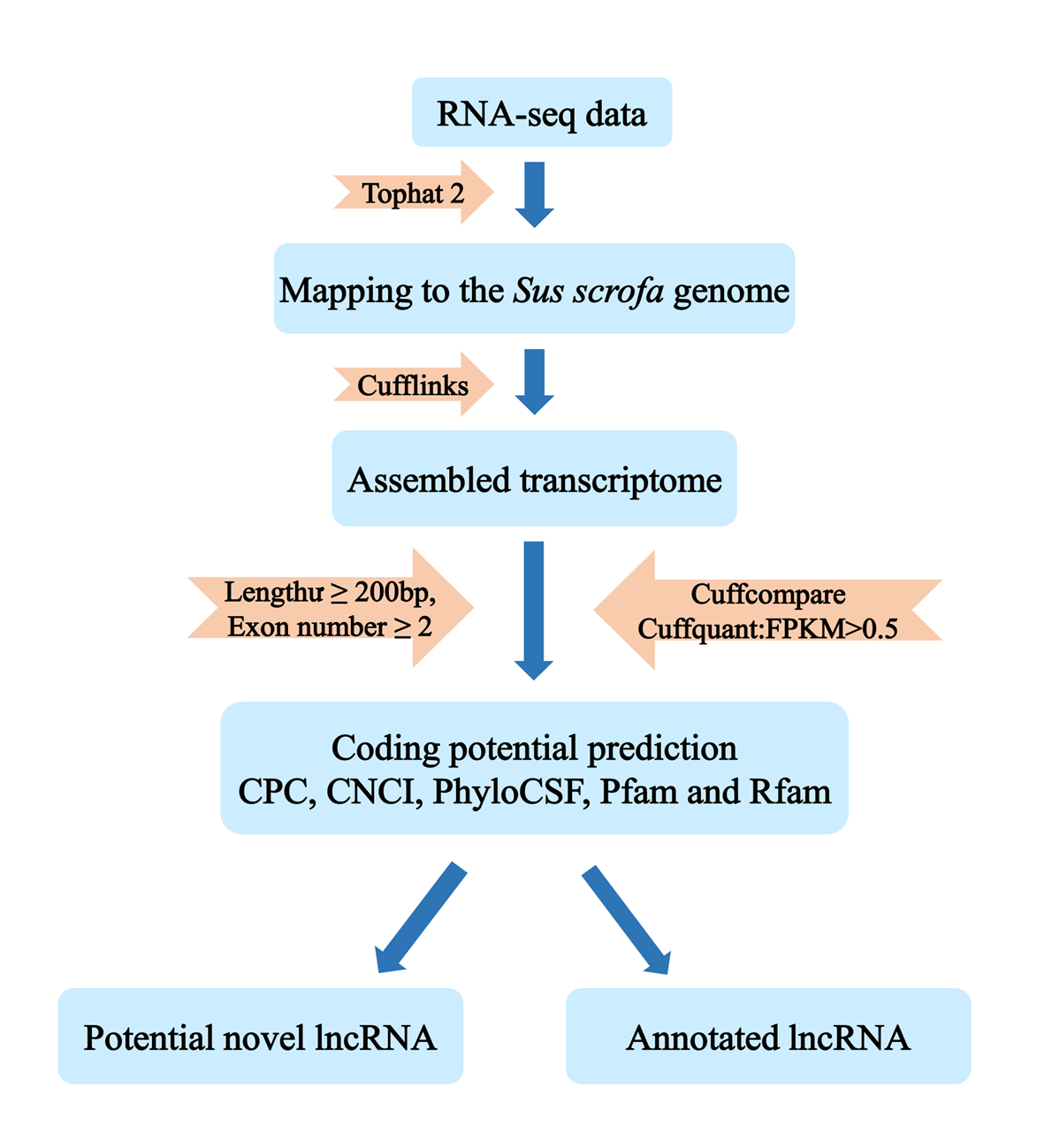

Supplement: Figure S2 — Identification pipeline for lncRNAs. Each step is described in detail in the methods section. [file Image_2.TIF]

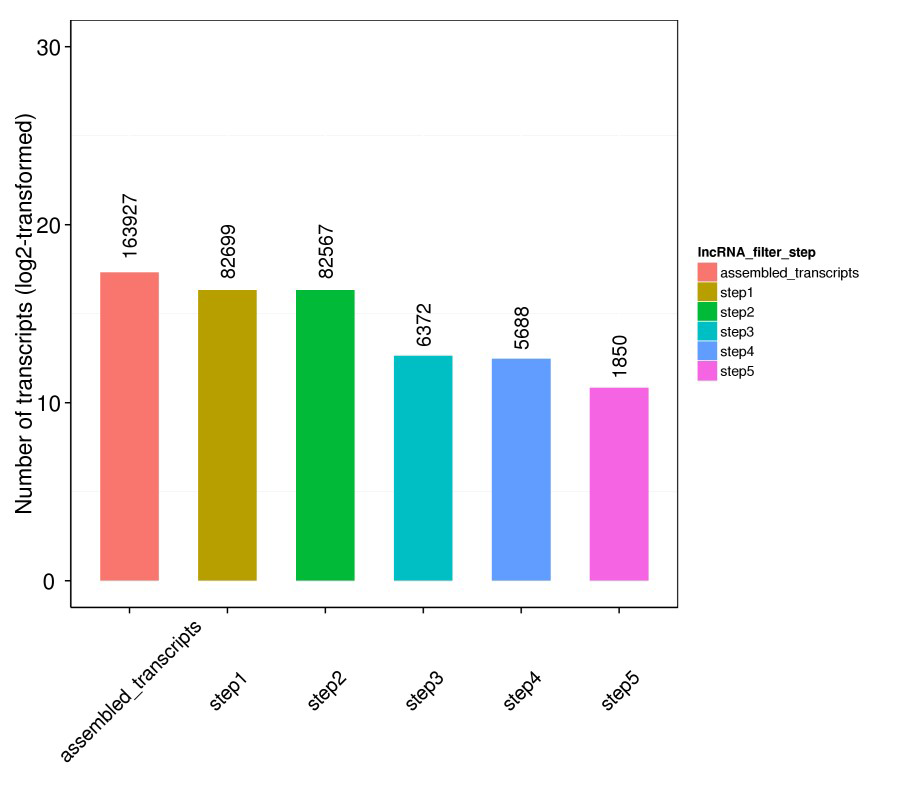

Supplement: Figure S3 — One thousand eight hundred and fifty non-coding lncRNAs were selected by Cufflinks and Scripture. [file Image_3.TIF]
